# Comparative and phylogenetic analysis of the complete chloroplast genome sequences of *Allium mongolicum*

**DOI:** 10.1038/s41598-022-26354-0

**Published:** 2022-12-15

**Authors:** Yanan Jin, Ting Zhang, Binke Liu, Chengzhong Zheng, Hongyan Huo, Jixing Zhang

**Affiliations:** 1 College of Life Science and Food Engineering, Inner Mongolia Minzu University, Tongliao, 028042 Inner Mongolia China; 2 Horqin Plant Stress Biology Research Institute of Inner Mongolia Minzu University, Tongliao, 012000 Inner Mongolia China; 3 Ulanqab Agricultural and Forestry Science Institute, Ulanqab, 028042 Inner Mongolia China

**Keywords:** Evolution, Molecular biology, Plant sciences

## Abstract

*Allium mongolicum* Regel is a wild and sandy vegetable with unique flavours. In this study, a complete chloroplast (cp) genome of *A. mongolicum* was obtained (Genbank accession number: OM630416), and contained 153,609 base pairs with the GC ratio as 36.8%. 130 genes were annotated including 84 protein-coding genes, 38 tRNA, and 8 rRNA genes. The large single-copy (LSC) region was 82,644 bp, and a small single-copy (SSC) region was 18,049 bp, which were separated by two inverted repeats (IRs, including IRa and IRb) of 26,458 bp. Comparative genome analyses of 55 *Allium* species suggested that genomic structure of genus *Allium* was conserved, and LSC and SSC regions were outstanding with high variability. Among them, more divergent loci were in the SSC region covering *ycf1*-*rrn4.5* and *ndhF*-*ccsA*. Phylogenetic analysis on cp genomes of 55 *Allium* determined that all members were clustered into 13 clades, and *A. mongolicum* had close relationship with *A. senescens*. Corresponding analyses of four protein-coding genes (*ycf1*, *ndhF*, *rpl32*, and *ccsA*) in aforementioned divergent loci confirmed that *ycf1* was finally chosen as the candidate gene for species identification and evolutionary classification of genus *Allium*. These data provide valuable genetic resources for future research on *Allium*.

## Introduction

*Allium mongolicum* Regel is famous for its unique flavour belonging to the genus *Allium* L. of Amaryllidaceae family. Combining the growth habitat and the same shape as green onions, it is called sandy onion. This species is naturally distributed in desert areas such as sandy land, gobi, arid hillside and dry river bed of China, Mongolia, Russia, Kazakhstan^1^. With strong drought-resistance, it is distributed from the northwest to northeastern of China (located between 36° 28′–46° 14′ N and 88° 39′–116° 05′ E) including Qinghai Province, Gansu Province, Shaanxi Province, Ningxia Hui Autonomous Region, Liaoning Province and Inner Mongolia Autonomous Region. In recent years, *A. mongolicum* has been widely planted in greenhouses after artificial domestication. Significantly, the *A. mongolicum* has greatly ornamental and ecological value^2^. Most studies have focused on nutrition and medicinal value^3,4^, and there has been no in-depth report of phylogenetic relationship with other *Allium* Species.

*Allium* is one of the largest and more important taxa in monocots, and covers multitudinous economical crops with unique nutritional and medicinal value, such as shallot, garlic, leek and onion^5^. However, the classification or phylogeny studies of *Allium* were controversial. Previous studies on classification of *Allium* had been mainly focused on the basis of subtle morphological difference, and some recent researches further classified the members by one or two loci such as internal transcribed spacer (ITS), *rps16,* and so on^6^. These results suggested *Allium* species can divide into 15 subgenera^7^. However, the phylogenetic studies of *Allium* have to be settled urgently.

Chloroplasts, originating from photosynthetic autotrophs of cyanobacteria, are unique independent organelles with a semi-autonomous genetic system, which can provide nutrients for plant growth and development. The complete chloroplast (cp) genomes are reliable elements for deducing phylogeny and evolutionary history deriving from highly conserved gene structure and gene content, and the cp genomes are short of recombination with low mutation rate^8^. Recently, many cp genomes have been extensively applied for phylogenetic reconstruction, and especially in *Allium* species^9–18^. However, these researches were carried out according to few members of *Allium* species. For example, sixteen cp genomes from *Cepa* were assessed the genes arrangement and variation, and a phylogeny of *Cepa* was constructed to explain the domestication of the common onion^19^. Xie et al. performed analyses of six cp genome structure, and uncovered the selective pressure in *Daghestanica*^20^. In addition, Xie et al. collected 39 *Allium* cp genomes and revealed that the divergence time of three traditional evolutionary lineages was presumably in the early Eocene to the middle Miocene^21^. However, excellent genes selected for reflecting classification and evolution of species in the genus *Allium* are scarce. Hence, the phylogenetic relationships from the point of total *Allium* species need to be further studied.

Here, high-throughput sequencing and bioinformatics technology were used to assemble a cp genome sequence of *A. mongolicum* (Genbank accession number: OM630416). 54 cp genomes from genus *Allium* were compared to explore the structure and evolution of the *A. mongolicum* chloroplast genome. Subsequently, phylogenomic analyses were performed based on these 55 chloroplast genomes to obtain a gene of choice for identifying and classifying of members from genus *Allium*. Our study will provide references for studying the genetic diversity and phylogenetic relationship in genus *Allium*.

## Results

### Basic characteristics of the *Allium mongolicum* chloroplast genome

**Figure 1:**
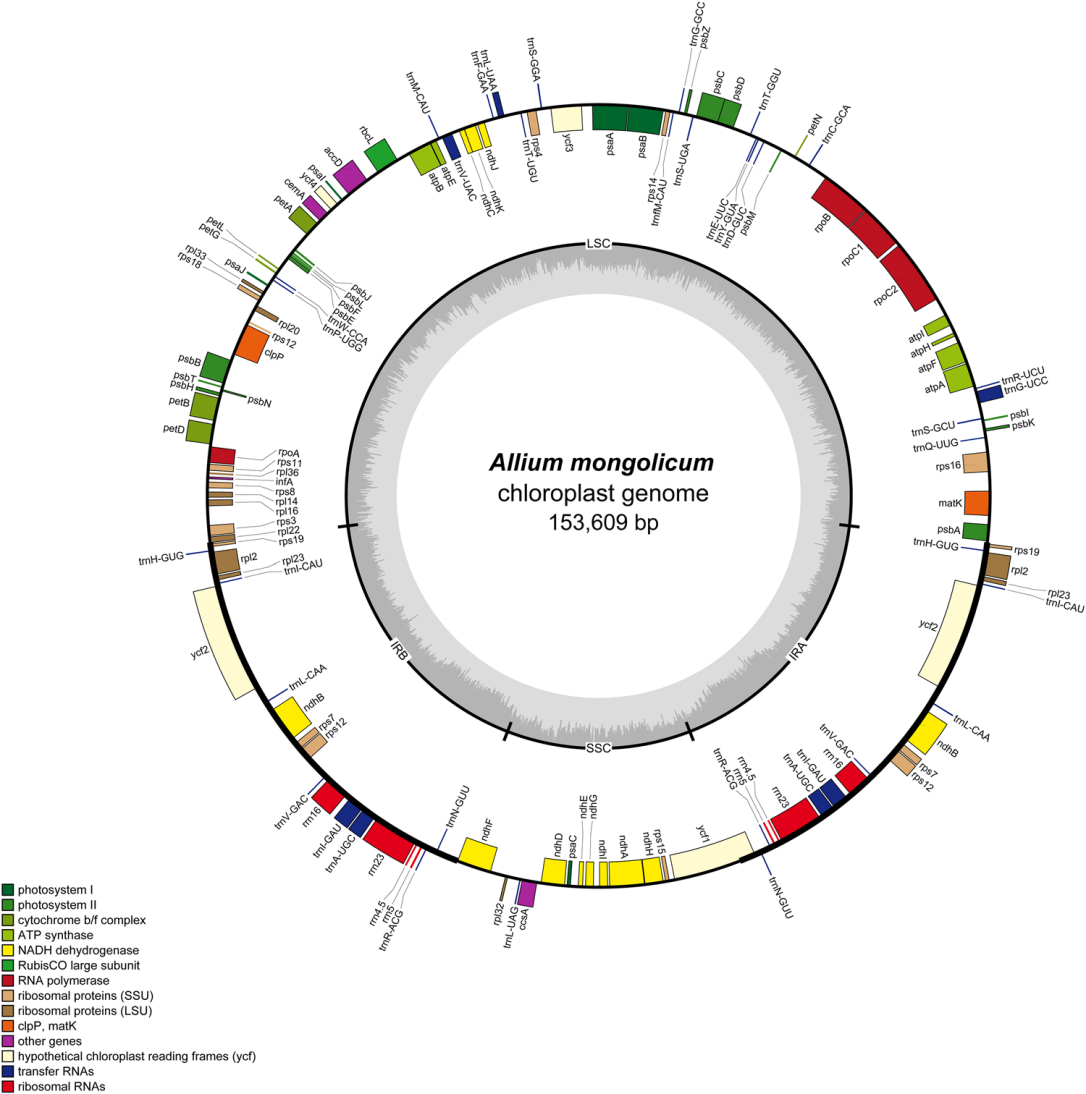
Gene structural map of *Allium mongolicum* chloroplast genome. Genes of different functional groups, Small single copy (SSC), Large single copy (LSC), and Inverted repeats (IRa, IRb), are separated by color. Genes drawn inside the circle are transcribed clockwise.

The extracted DNA was assessed, and the initial raw data with 8.98 Gbp was used to subsequent analysis. The finished cp genome was submitted to GenBank with the accession number OM630416. The length of the cp genome of the *A. mongolicum* was 153,609 bp, composed of a large single-copy (LSC) region of 82,644 bp and a small single-copy (SSC) region of 18,049 bp separated by two inverted repeats (IRs, including IRa and IRb) of 26,458 bp (Fig. 1). The overall GC (guanine and cytosine) content of the whole chloroplast genome was 36.8%. A total of 130 genes were annotated, including 84 protein-coding genes, 38 tRNA, and eight rRNA genes (Table 1). There are 21 chloroplast genes harbored introns, among which 19 genes contained single introns, and two genes (*ycf3*, *clpP*) contained two introns.

**Table 1:** Genes contained in *Allium mongolicum* chloroplast genome.

Category	Groups of genes	Name of genes
Self-replication	Ribosomal RNA	*rrn4.5*^*b*^, *rrn5*^*b*^, *rrn16*^*b*^, *rrn23*^*b*^
	Transfer RNA	*trnA-UGC*^*a,b*^, *trnC-GCA*, *trnD-GUC*, *trnE-UUC*, *trnF-GAA*, *trnfM-CAU*, *trnG-GCC*, *trnG-UCC*^*a*^, *trnH-GUG*^*b*^, *trnI-CAU*^*b*^, *trnI-GAU*^*a,b*^, *trnL-CAA*^*b*^, *trnL-UAA*^*a*^, *trnL-UAG*, *trnM-CAU*, *trnN-GUU*^*b*^, *trnP-UGG*, *trnQ-UUG*, *trnR-ACG*^*b*^, *trnR-UCU*, *trnS-GCU*, *trnS-GGA*, *trnS-UGA*, *trnT-GGU*, *trnT-UGU*, *trnV-GAC*^*b*^, *trnV-UAC*^*a*^, *trnW-CCA*, *trnY-GUA*
	Ribosomal proteins	*rps3*, *rps4*, *rps7*^*b*^, *rps8*, *rps11*, *rps12*^*a,b*^, *rps14*, *rps15, rps16*^*a*^, *rps18*, *rps19*^*b*^, *rpl2*^*a,b*^, *rpl14*, *rpl16*, *rpl20*, *rpl22*, *rpl23*^*b*^, *rpl32*, *rpl33*, *rpl36*
	Transcription	*rpoA*, *rpoB*, *rpoC1*^*a*^, *rpoC2*
Photosynthesis related genes	Photosystem I	*psaA*, *psaB*, *psaC*, *psaI*, *psaJ*
	Assembly/stability of photosystem I	*ycf3*^*a*^, *ycf4*
	Photosystem II	*psbA*, *psbB*, *psbC*, *psbD*, *psbE*, *psbF*, *psbH*, *psbI*, *psbJ*, *psbK*, *psbL, psbM*, *psbN*, *psbT*, *psbZ*
	NADPH dehydrogenase	*ndhA*^*a*^, *ndhB*^*a,b*^, *ndhC*, *ndhD*, *ndhE*, *ndhF*, *ndhK*, *ndhG*, *ndhH*, *ndhI*, *ndhJ*
	ATP synthase	*atpA*, *atpB*, *atpE*, *atpF*^*a*^, *atpH*, *atpI*
	Cytochrome b/f compelx	*petA*, *petB*^*a*^, *petD*^*a*^, *petG*, *petL*, *petN*
	Rubisco	*rbcL*
Other genes	Others	*accD*, *ccsA*, *cemA*, *clpP*^*a*^, *infA*, *matK*, *ycf1*, *ycf2*^*b*^

^a^Intron-containing genes. ^b^Genes located in the IR regions.

### Characteristics and comparison of *Allium* cp genomes

To further explore the differences among the cp genome sequences from genus *Allium*, 54 reported cp genomes of *Allium* were compared to the cp genome sequence of *A. mongolicum*. The detailed information of all cp genomes was listed in the Table 2. Results showed that these genomes sequenced were similar with size and structure. The length of these genomes was generally 145,819–154,804 bp (*A. paradoxum* and *A. monanthum*, respectively) including LSC region (72,386–83,835 bp), SSC region (13,504–21,706 bp) and two IR regions (24,561–26,924 bp). *A. monanthum* possessed the longest cp genome in length, and this may be on account of the longest LSC region with 83,835 bp. Similarly, ungrouped *A. paradoxum* had the shortest cp genome in length with the shortest SSC region (13,504 bp). However, *A. trifurcatum* contained the longest SSC region and the shortest IR regions, *A. tenuissimum* accompanied by the shortest SSC region, *A. cernuum* was together with the longest IR region, and the three ones in length were not extreme.

**Table 2:** Basic features of chloroplast genomes in *Allium.*

Species	Accession Number	LSC	IRb	SSC	IRa	Length
*A. nanodes*	NC_045520	82,978	26,540	17,974	26,540	154,032
*A. ovalifolium*	MK628479	82,683	26,505	17,932	26,505	153,625
*A. prattii*	NC_037432.1	83,428	26,575	17,904	26,575	154,482
*A. tricoccum*	NC_057583	82,606	26,531	17,897	26,531	153,565
*A. victorialis*	NC_037240.1	83,169	26,526	17,853	26,526	154,074
*A. cyathophorum*	NC_045912	83,359	26,467	17,881	26,467	154,174
*A. hookeri*	MZ557488	82,609	26,798	17,387	26,798	153,592
*A. trifurcatum*	NC_045844	82,628	24,561	21,706	24,561	153,456
*A. neriniflorum*	NC_049103	83,131	26,479	18,191	26,479	154,280
*A. nigrum*	NC_057585	81,636	26,367	17,871	26,367	152,241
*A. cepa*	MK335926.1	82,719	26,468	17,931	26,468	153,586
*A. galanthum*	NC_050981	82,402	26,466	17,888	26,466	153,222
*A. caeruleum*	NC_049099	82,389	26,410	18,058	26,410	153,267
*A. chinense*	NC_043922.1	81,323	26,498	18,206	26,498	152,525
*A. monanthum*	NC_042726.1	83,835	26,481	18,007	26,481	154,804
*A. oschaninii*	NC_044470.1	82,521	26,514	18,031	26,514	153,580
*A. praemixtum*	NC_044412.1	82,162	26,511	18,042	26,511	153,226
*A. pskemense*	NC_044411.1	82,720	26,517	18,034	26,517	153,788
*A. schoenoprasoides*	NC_049105	82,551	26,475	18,082	26,475	153,583
*A. fetisowii*	NC_049100	83,657	26,210	17,941	26,210	154,018
*A. karataviense*	NC_057573	81,804	26,368	17,268	26,368	151,808
*A. ampeloprasum*	NC_044666.1	81,774	26,526	17,906	26,526	152,732
*A. ferganicum*	NC_057975	81,981	26,556	18,033	26,556	153,126
*A. sativum*	MK335928.1	82,012	26,564	18,049	26,564	153,189
*A. changduense*	MK299426	82,906	24,642	21,469	24,642	153,659
*A. chrysocephalum*	NC_042155.1	82,688	26,512	17,998	26,512	153,710
*A. cyaneum*	MK299427	83,829	25,117	18,167	25,117	152,230
*A. forrestii*	NC_049101	82,339	26,455	17,937	26,455	153,186
*A. kingdonii*	NC_046030	83,423	26,163	17,810	26,163	153,559
*A. koreanum*	NC_057579	82,216	26,530	17,886	26,530	153,162
*A. maowenense*	NC_042157.1	82,668	26,470	18,000	26,470	153,608
*A. mongolicum*	OM630416	82,644	26,458	18,049	26,458	153,609
*A. nerinifolium*	NC_057574	82,871	26,542	18,174	26,542	154,129
*A. obliquum*	NC_037199.1	81,588	26,370	18,059	26,370	152,387
*A. paepalanthoides*	MK299429	82,716	26,331	17,957	26,331	153,335
*A. plurifoliatum*	MK299430	82,962	25,818	18,636	25,818	153,234
*A. polyrhizum*	NC_049104	82,438	25,893	18,862	25,893	153,086
*A. przewalskianum*	MN519210	82,302	26,745	17,717	26,745	153,509
*A. ramosum*	MH159131	83,090	26,519	17,906	26,519	154,034
*A. rude*	NC_042158.1	82,815	26,452	17,978	26,452	153,697
*A. senescens*	NC_057580	82,567	26,491	17,993	26,491	153,542
*A. sikkimense*	MK299431	81,738	26,470	17,871	26,470	152,549
*A. spicatum*	NC_045911	82,625	26,321	17,920	26,321	153,187
*A. strictum*	MK820622	82,880	25,759	18,564	25,759	152,962
*A. tenuissimum*	MW759301	72,386	26,492	20,871	26,492	146,241
*A. tuberosum*	NC 044,709.1	83,068	26,515	17,958	26,515	154,056
*A. xichuanense*	NC_042159.1	82,797	26,463	17,950	26,463	153,673
*A. altaicum*	NC_040972.1	82,196	26,510	17,913	26,510	153,129
*A. chrysanthum*	MH992108.1	82,744	26,446	17,985	26,446	153,621
*A. fistulosum*	MK335927.1	82,235	26,510	17,907	26,510	153,162
*A. schoenoprasum*	NC_057575	81,888	26,515	17,934	26,515	152,852
*A. cernuum*	NC_057572	82,294	26,924	17,608	26,924	153,750
*A. paradoxum*	MH053150.1	80,919	25,698	13,504	25,698	145,819
*A. siculum*	NC_057584	83,308	26,436	18,045	26,436	154,225
*A. ursinum*	MH157875.2	82,086	26,895	17,376	26,895	153,252

**Figure 2:**
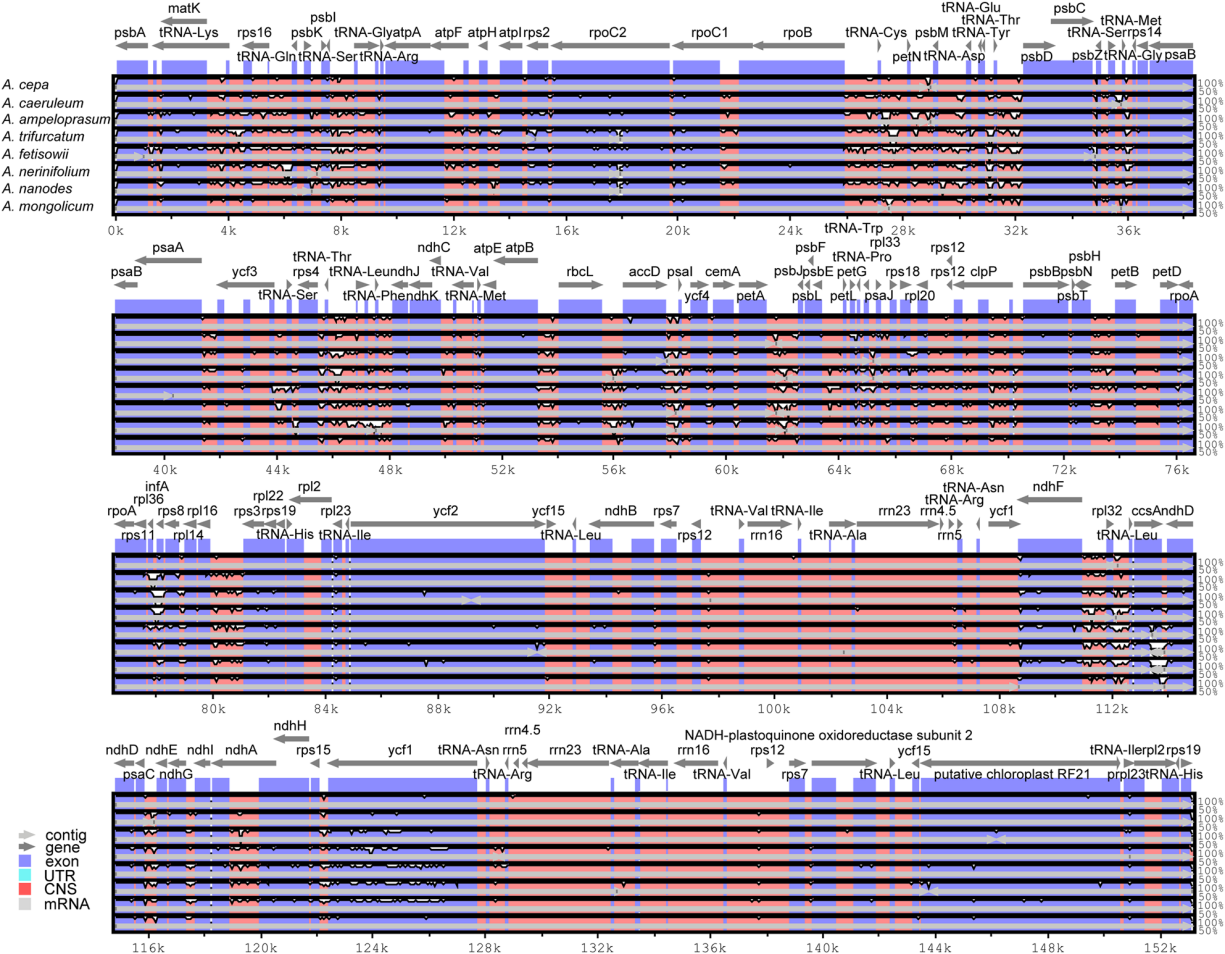
Sequence alignment of 9 representative *Allium* species using in mVISTA. The Y-scale represents the percentage of identity ranging from 50 to 100%.

In order to elucidate characteristics of sequence divergence among the 55 *Allium* cp genomes, nine representative species were selected to estimate *Allium* genome comparability using mVISTA program. The genome of *A. altaicum* was taken as the reference to conduct this program. Results revealed that cp genomes were relatively conserved in all *Allium* genomes (Fig. 2). No notably differences in gene order were detected when comparing the *A. mongolicum* cp genome to those of related *Allium* species. Generally, the highly divergent regions among representative *Allium* cp genomes mainly occurred in the LSC and SSC regions, and the coding regions exhibited less variations than the non-coding regions. In terms of species, high similarity and low divergence were detected in the cp genomes among *A. altaicum*, *A.cepa* and *A. mongolicum*, and the same things were tested among the group covering *A. caeruleum* and *A. ampeloprasum*, as well as group including *A. trifurcatum*, *A. fetisowii*, *A. nerinifolium* and *A. nanodes*.

**Figure 3:**
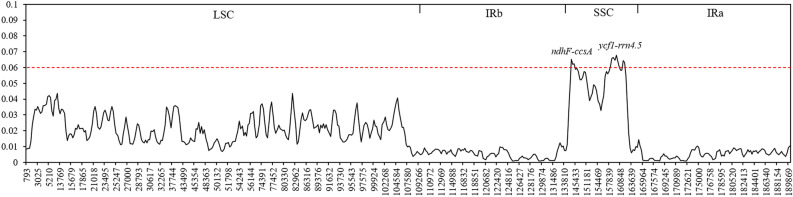
Comparative analysis of the nucleotide variability (*Pi*)values of 55 *Allium* species.

To further exploit available polymorphic genes for identifying novel species, we further calculate the nucleotide diversity (*Pi*) values of overall sequences within the 600 bp window (Fig. 3). We found that *Pi* values ranged from 0 to 0.06769, and relatively high *Pi* values were determined in the SSC region, followed closely by those in the LSC region. The average *Pi* values of SSC and LSC regions were 0.0456 and 0.0219, respectively, but that of IR regions was 0.0052 (Table S1). These differences showed that two IR regions were more conserved than LSC and SSC regions. Here, most of cp genomes variations of *Allium* species existed in the SSC region, and two hypervariable regions (*Pi* > 0.06) were highlighted including variable loci *ycf1*-*rrn4.5* (0.06052–0.06769) and *ndhF*-*ccsA* (0.06195–0.06516). The divergent regions called *ycf1*-*rrn4.5* contained *ycf1*, *tRNA-Asn*, *tRNA-Arg*, *rrn5* and *rrn4.5*, and *ndhF*-*ccsA* loci comprised *ndhF*, *rpl32*, *tRNA-Leu* and *ccsA*. Among these genes, four coding genes (*ycf1*, *ndhF*, *rpl32*, and *ccsA*) were understanding in terms of general conservation. Thus, these polymorphic regions might be novel candidate fragments for population genetic studies of *Allium* species.

### Codon usage

**Figure 4:**
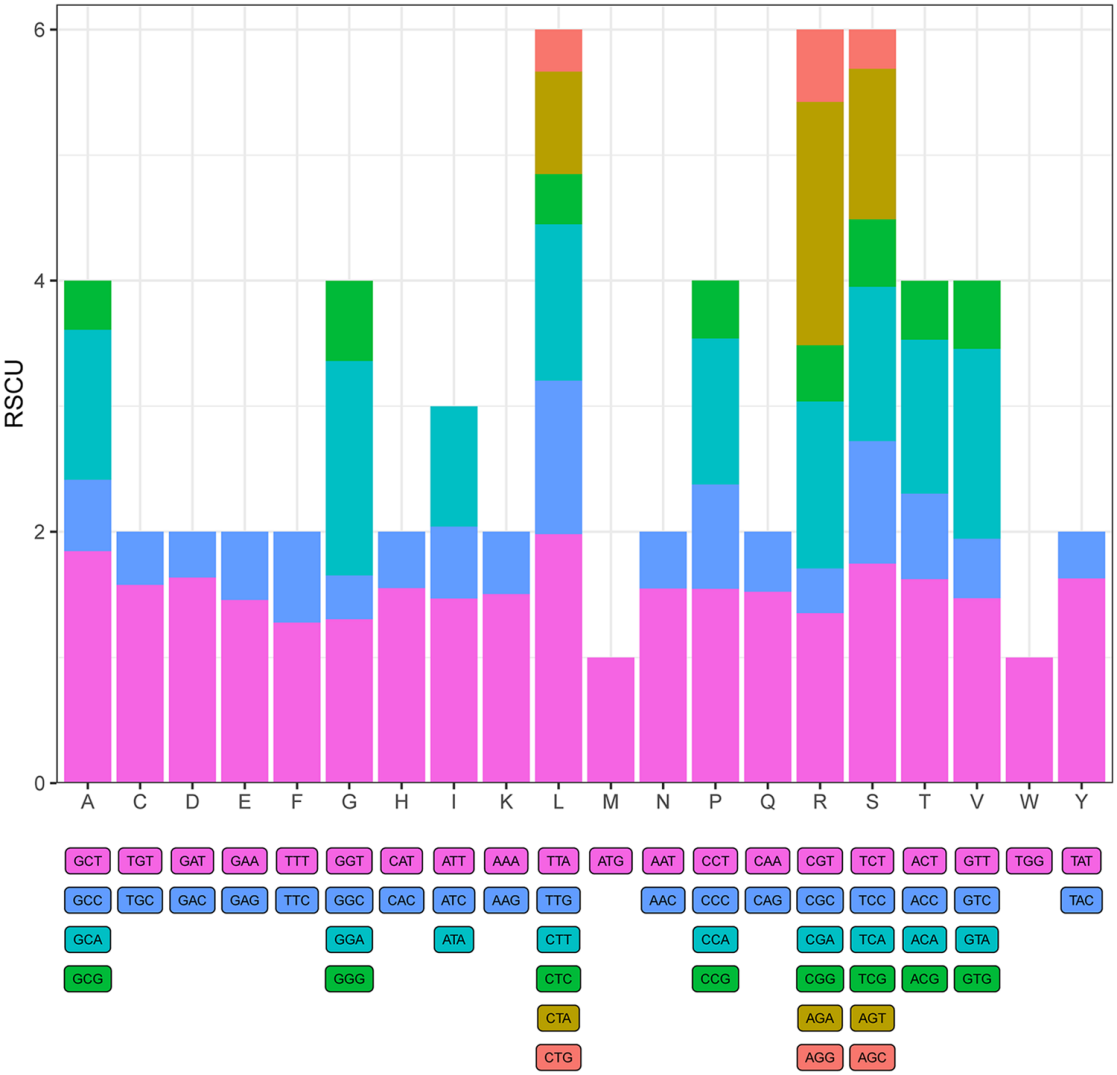
The values of relative synonymous codon usage (RSCU) of *Allium mongolicum*.

Codon usage is the correlation to mRNA and protein, which is an essential characteristic for gene expression in plants genomes^12^. We estimated in detail codon usage frequency associated with all protein-coding sequences in *A. mongolicum*. Results showed that leucine (L), arginine (R) and serine (S) were the highest frequent codons, and methionine (M) and tryptophan (W) were least frequent (Fig. 4). Next, 51 grouped *Allium* species cp genomes were used to calculated in detail codon usage frequency, and results showed that these protein-coding genes were encoded by 19,832 (*A. przewalskianum*) to 26,802 (*A. fistulosum*) codons (Table S2). Like to other monocots plants, UGA, UAG, and UAA were known as the termination codons. For these *Allium* species, we found that the UUA encoded leucine (Leu) had the highest relative synonymous codon usage (RSCU) value in *A. przewalskianum* at approximately 2.23, and the in *A. przewalskianum* CUG encoded leucine had the lowest RSCU value at approximately 0.29. Same to *A. mongolicum*, methionine and tryptophan are used the least, but only leucine is used the most. In addition, 30 codons of *Allium* species were > 1, and 32 codons were < 1. Similar to other monocots chloroplast genomes, the nucleotide frequency of G or C from *Allium* species was lower than those of A or T at the whole codon, as well as the third codon position. The preference was considered to be a comprehensive result of gene expression, natural selection, and speciation mechanisms in species^19^.

### Contraction and expansion of IR regions

**Figure 5:**
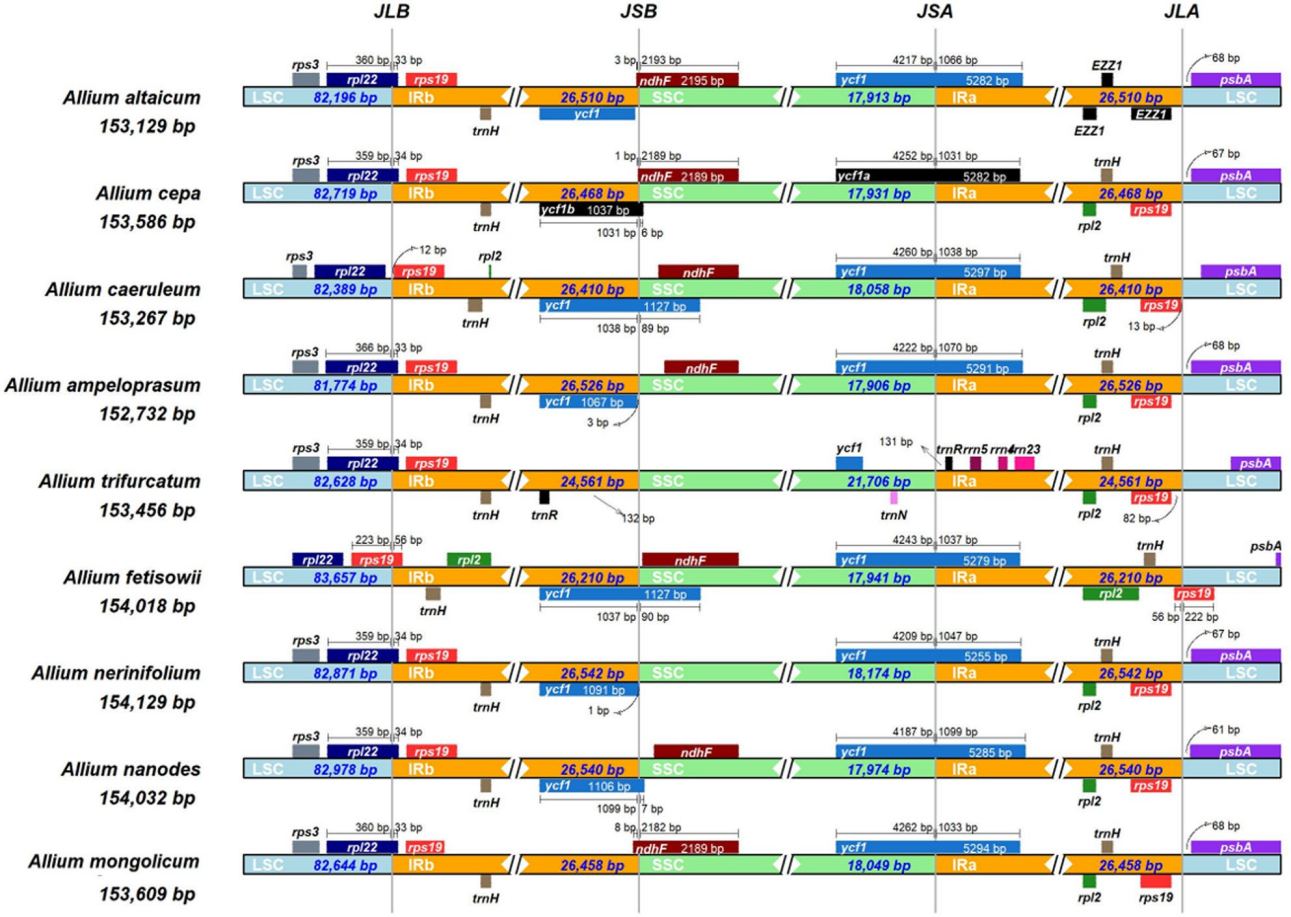
Comparison of the junctions between the LSC, SSC and IR regions among 9 representative *Allium* chloroplast genome.

It is well known that the cp genomes are usually well conserved with typical quadripartite structure and specific order. Through diversities of cp genomes mainly depend on highly divergent and lowly variable LSC and SSC regions, the size variations of cp genomes are strongly linked to the contraction and expansion of two IR regions, which can reflect species evolution. Therefore, IR boundaries of 9 representative members were detected to explain the differences of *Allium* cp genome size, including the boundaries of LSC/IRb regions (JLB), boundaries of SSC/IRb regions (JSB), boundaries of SSC/IRa regions (JSA), and boundaries of LSC/IRa regions (JLA). Results exhibited that the boundaries of different regions in various *Allium* species were different (Fig. 5). Generally, JLB were found to be located within the *rpl22* gene up to a distance of 359–366 bp except for *A. caerulrum* (none) and *A. fetisowii* (*rps19*), and this partial expansion of the IRb region of *A. fetisowii* may result in the longest LSC regions (83,657 bp). JSB from three species (*A. altaicum*, *A. cepa*, *A. mongolicum*) was located in the *ndhF* gene, and those of four species was *ycf1* gene. JSA in the seven cp genomes was located in the *ycf1* gene. JLA were found between *rps19* gene and *psbA* gene. Interesting, *A. mongolicum* cp genome has lost the *ycf1* gene on the JSB. Overall, four connections of *A. mongolicum* had nearly identical relative positions with *A. nanodes*, *A. ampeloprasum*, and *A. cepa*.

### Phylogenetic analysis

**Figure 6:**
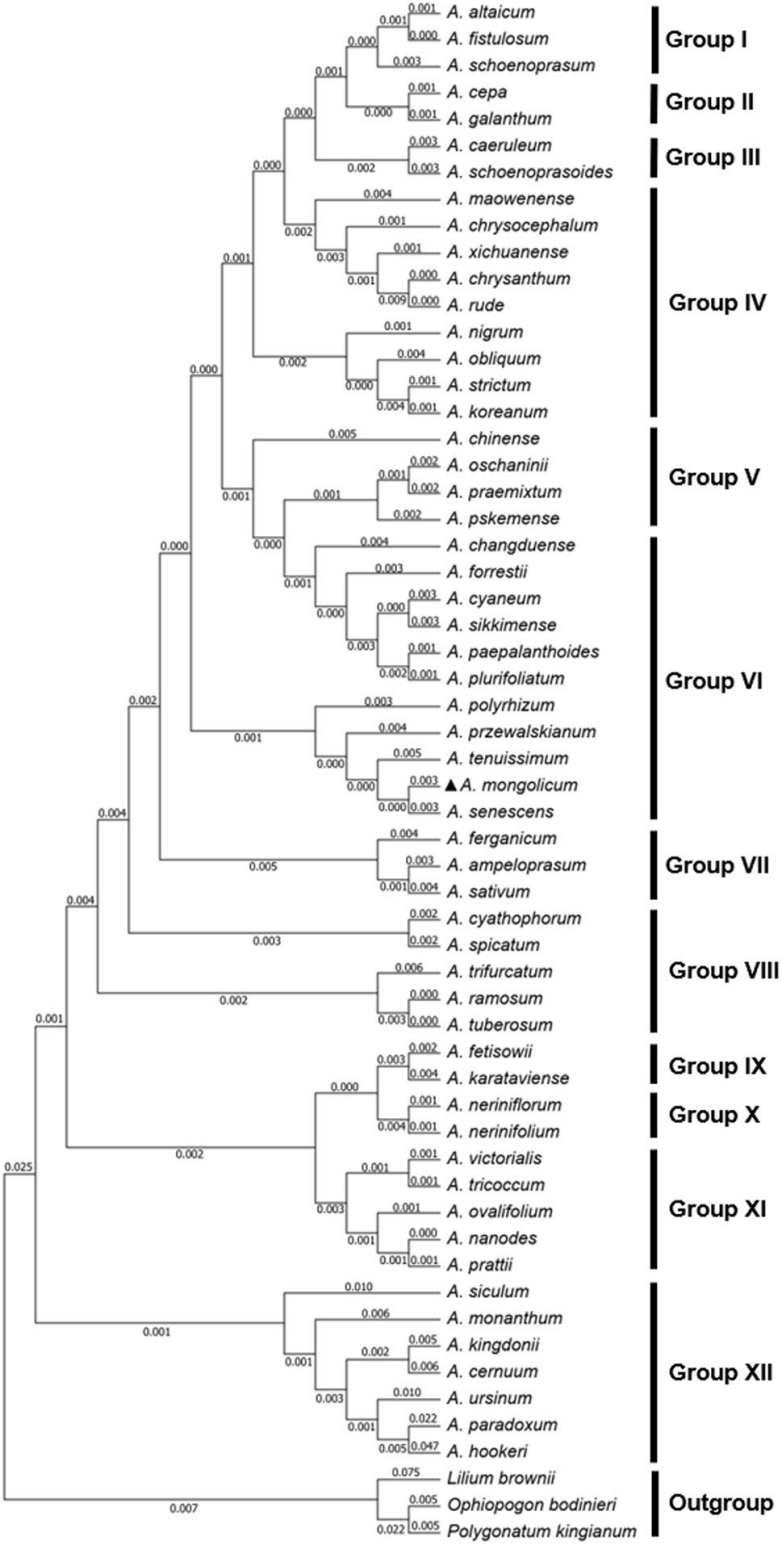
Phylogenetic relationships of 55 *Allium* species using Maximum Likelihood (ML) analyses, and *Lilium brownie* (MK493294), *Ophiopogon bodinieri* (NC_051508) and *Polygonatum kingianum* (MW373520) were used as the outgroup.

**Figure 7:**
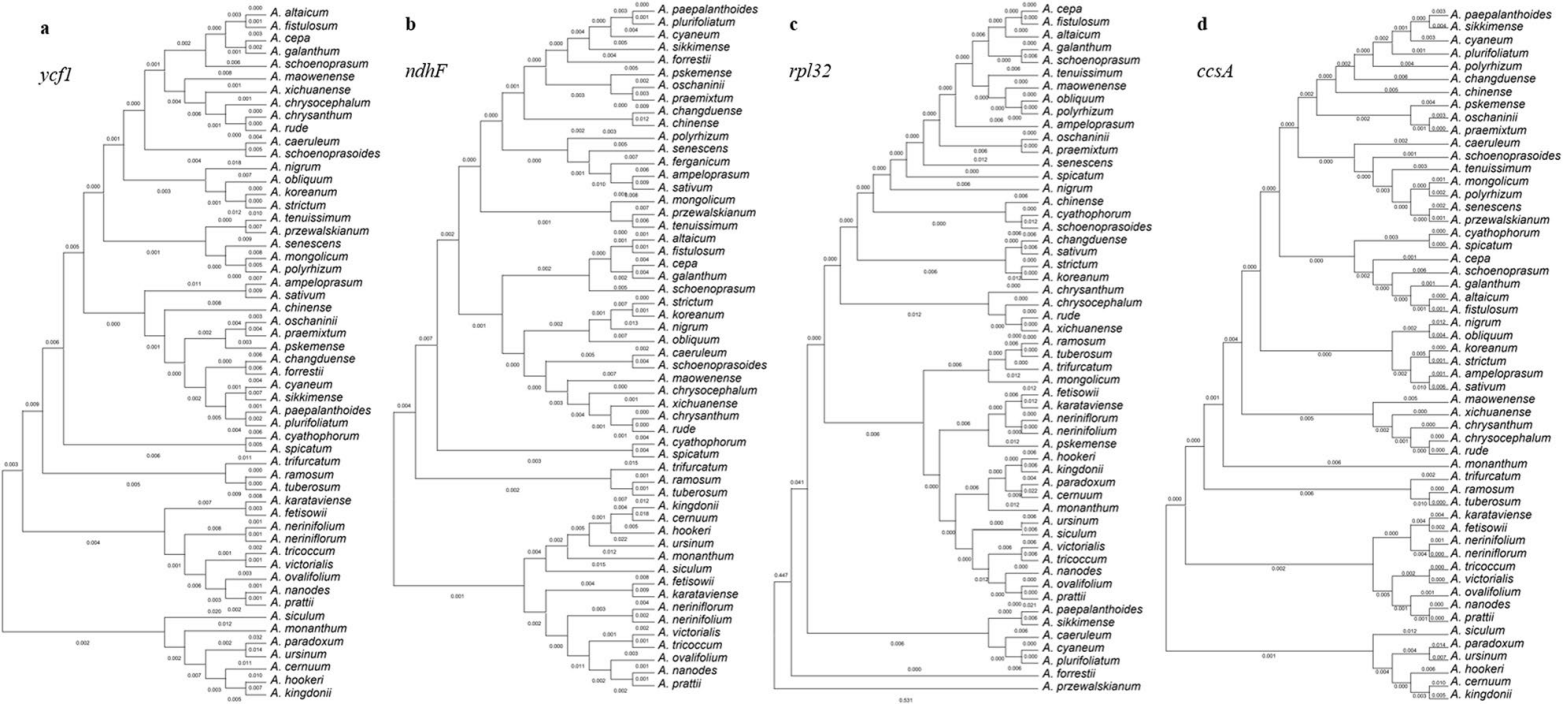
Phylogenetic relationships of four protein-coding genes from 55 *Allium* species using Maximum Likelihood (ML) analyses. (**A**) *ycf1* gene; (**B**) *ndhF* gene; (**C**) *rpl32* gene; (**D**) *ccsA* gene.

To know the evolutionary location of *A. mongolicum* and genetic clusters of genus *Allium*, phylogenetic tree of 55 *Allium* members was constructed. Phylogenetic analysis of the cp genome suggested that all members were clustered into 13 clades, and three outgroup ones derived from one clade (Fig. 6). Among them, *A. mongolicum* found in this study had close relationship with *A. senescens* in the genus *Allium*. To further obtain a gene of choice on assessing phylogeny of *Allium* species based on aforementioned *Pi* values, we constructed the phylogenetic trees according to the most variable four protein-coding genes (*ycf1*, *ndhF*, *rpl32*, and *ccsA*). Results showed that these phylogenetic trees were less similar with various major clades, and the cladogram for *ycf1* was accordant to those of the whole-length cp genomes (Fig. 7).

## Discussion

*Allium mongolicum* is distributed in desert areas with strong drought-resistance, and is an infrequent *Allium* species of Amaryllidaceae family. Unlike other *Allium* plants, *A. mongolicum* mainly located in the barren land with territorial restrictions, and related researches were lacking. As an essential member of genus *Allium*, it plays a critical role in explaining the evolutionary relationship of genus *Allium*. In this study, we sequenced a cp genome of *A. mongolicum* (153,609 bp), and exhibited the typical quadripartite structure including 130 unigenes. These genes were composed of 84 coding genes, 38 tRNAs and 8 rRNAs. Compared to cp genomes from 55 genus *Allium*, all genomes had a high conservation in the genome structure, length and organization. It is obvious that the LSC and SSC regions were highly divergent regions than two IR regions, and analysis of nucleotide diversity also confirmed this view. We further found that the more variable loci were in the SSC region comprising *ycf1*-*rrn4.5* and *ndhF*-*ccsA*. The traditional taxonomy of *Allium* is mostly based on plant morphology such as tillering characteristics and pseudo-stem morphology. For a long time, the taxonomic status of *Allium* genus has changed frequently. In this study, phylogenetic analysis of full-length cp genome revealed that 55 *Allium* members were separated into 13 clades. In addition, members from Group VI including *A. mongolicum* shared closer relationship and more similar gene contents with members from Group VII. The 13 clades obtaining from the phylogenetic analyses of cp genomes was in accord with the modern taxonomic classification. In addition, the detailed evolutionary analyses of four protein-coding genes from two above-mentioned key loci were performed, and *ycf1* was finally chosen as the candidate gene for species identification and evolutionary classification of genus *Allium*.

Contrastive analysis of the *Allium* chloroplast genomes showed that the size was wide range from 145 to 154 kb, and the difference was mainly due to JSB and JSA regions. Two *ycf1* genes on the boundaries of IR regions were crucial, which concatenated the single copy region and the reverse repeat region. Specifically speaking, the length of *A. monanthum* was longest, and two *ycf1* genes of those were longer with 1130 bp and 5309 bp, respectively. On the side, two *ycf1* genes of *A. paradoxum* were severally 521 bp and 5249 bp, resulting in the shortest cp size. This phenomenon was coincident with the studies of comparative chloroplast genomics of the genus *Taxodium*^22^, and the changes in length of the two genes brought out the reduction and expansion of two IR regions, which directly impacted the size of the chloroplast genome.

Further characteristics of *Allium* sequence divergence and diversity were analyzed through multiple ways in this study, and all results referred to the single copy regions were more variable than two IR regions. Interestingly, *ycf1*-*rrn4.5* and *ndhF*-*ccsA* in the SSC region were the most clustered diversity sites in the whole chloroplast genome of genus *Allium*. These two sites contained 4 protein coding-genes (*ycf1*, *ndhF*, *rpl32*, and *ccsA*) and 5 non-coding genes (tRNA-Leu, tRNA-Asn, tRNA-Arg, rrn5 and rrn4.5). Researcher had reported that *rpoC2* can be taken as a gene of choice in *Allium* phylogeo-graphical studies, but the results were pointed out by the phylogenetic tree of 17 members of genus *Allium*. Hence, digging the best candidate gene for genus *Allium* evolution analysis was extremely urgent. The phylogenetic tree of 55 *Allium* species was constructed from chloroplast genome sequences, and further evolution analyses of four variable protein-coding genes in all species were performed. Results suggested that the evolutionary relationship of *ycf1* was consistent in the tree of the whole-length cp genomes. Hence, *ycf1* was the best choice for assessing classification of *Allium* species.

## Conclusions

In this study, we successfully provided a complete chloroplast genome of *A. mongolicum* with 130 genes. Compared to genome structure with other members from *Allium* genus, the size, structure, gene contents of *A. mongolicum* chloroplast genome was conserved. Fifty-five *Allium* species in total were used to comparative analysis, and the phylogenetic analysis using full-length genome sequence were in accordance with the results using highly diverse *ycf1* gene. Hence, *ycf1* gene can be employed to evaluate phylogenetic relationships in *Allium*. The molecular data in this study provide a valuable resource for the study of evolution in *Allium* genus.

## Materials and methods

### Sampling, DNA extraction

*Allium mongolicum* seeds in this study were obtained from National Wild Plant Germplasm Resource Center, Kunming, Yunnan, China (Number: 178291), and was preserved at Horqin Plant Stress Biology Research Institute of Inner Mongolia Minzu University (Tongliao, China). The study protocol complied with relevant institutional, national, and international guidelines and legislation. The genomic DNA was extracted by the CTAB method^23^ assessing by the values of OD_260/280_.

### Chloroplast genome sequencing, assembly and annotation

The qualified DNA was fragmented and used to construct the library, then sequenced on an Illumina Hiseq platform by two-terminal sequencing strategy. Firstly, the initial raw data was controlled and plotted by FastQC (http://www.bioinformatics.babraham.ac.uk/projects/fastqc/), seqtk (1.3-r106), fastp (0.19.5) and R (3.6.1). Then, software SPAdes v3.61 under different K-mer parameters were used for contig assembly^24^. Finally, software CPGAVAS2, Dual Organellar GenoMe Annotator (DOGMA, http://dogma.ccbb.utexas.edu/) and blast + (2.9.0) were used for genes annotation. The finished cp genome was submitted to GenBank (OM630416).

### Genome comparison

Fifty-four reported cp genomes of genus *Allium* were downloaded from National Center for Biotechnology Information (NCBI, https://www.ncbi.nlm.nih.gov), and the detailed information was listed in the Table 2. The divergence of 9 representative genomes was operated by mVISTA (https://genome.lbl.gov/vista/index.shtml) in Shuffle-LAGAN mode^25,26^. MAFFT program was used to align all *Allium* species cp genomes^27^. *Pi* of all cp genomes was counted using Launch DnaSP6^28^, and results were presented through a sliding window analysis with a window length of 600 bp and step size of 200 bp. Boundaries of IR regions, contraction and expansion of cp genomes were visualized by Program IRscope^29^.

### Codon usage analysis

We selected Program codonW 1.4.4 to obtain the values of RSCU for evaluating codon preference^30^.

### Phylogenetic analysis

A phylogenetic tree of 55 genus *Allium* members was constructed using MEGA-X through Maximum likelihood (ML) method^31^. Three related species were adopted as the outgroup containing *Lilium brownie* (MK493294), *Ophiopogon bodinieri* (NC_051508) and *Polygonatum kingianum* (MW373520). Phylogenetic trees of four targets genes (*ndhF*, *rpl32*, *ccsA* and *ycf1*) were constructed by the same way.

## Supplementary Information

Supplementary Information 1.

Supplementary Information 2.

## Data Availability

The datasets generated during the current study are available in the NCBI repository (Genbank accession number: OM630416).
